# The Impact of Educational Intervention on the Anxiety of Family Caregivers of the Elderly with Dementia: A Randomized Controlled Trial

**DOI:** 10.30476/ijcbnm.2020.81680.0

**Published:** 2020-07

**Authors:** Abdolrahman Zarepour, Maryam Hazrati, Ali Akbaar Kadivar

**Affiliations:** 1 Student Research Committee,Shiraz University of Medical Sciences, Shiraz, Iran; 2 Department of Nursing, School of Nursing and Midwifery, Shiraz University of Medical Sciences, Shiraz, Iran; 3 Department of Neurology, school of Medicine, Shiraz University of Medical Sciences, Shiraz, Iran

**Keywords:** Anxiety, Dementia, Education, Family caregivers

## Abstract

**Background:**

Due to the increasing population of elderly and the consequent increase in the number of chronic diseases such as dementia, the psychological complications such as anxiety in the family caregivers increase. The aim of this study was to determine the effect of educational intervention on the anxiety of family caregivers of the elderly people with dementia.

**Methods:**

This randomized controlled trial was performed in the elderly Neurology Clinics in Shiraz from May to August 2017. This study was conducted on 70 families with elderly people with dementia who were randomly divided into an intervention (receiving in groups for seven sessions of educational intervention) and a control group (Conventional care). Data collection tool was Spielberger’s Anxiety Inventory (40-items, score=20-80). Data were analyzed by SPSS version 21 using ANOVA test with repeated measures and independent t-test.

**Results:**

One and three months after the interventions, the mean scores of anxiety in the intervention group were 70.51±3.78 and 70.31±3.43 and in the control groups they were 76.45±3.45 and 76.22±5.08 respectively. The results showed significant differences between the two groups regarding anxiety after the intervention (P<0.001).

**Conclusion:**

Educational programs held to promote and maintain the physical and mental health of caregivers could reduce the anxiety of family caregivers of the elderly with dementia.

**Trial Registration Number:** IRCT2017080915426N4.

## INTRODUCTION

According to the World Health Organization (2016), 47.5 million people worldwide suffer from dementia and it will reach 76 million by 2030. ^1 , 2^ Precise statistics on the prevalence of dementia in Iran are not available. It is estimated that over 500,000 people in Iran suffer from Alzheimer’s type of dementia. ^3^ Elderly diseases, such as dementia, are expected to increase as age grows. ^4^ Dementia is a long-term, progressive syndrome ^1^ in which the level of consciousness in the individual does not change and causes disruptions in behavior, ability to perform daily activities, thinking and memory. ^5 , 6^ In this case, there is a sharp decrease in the elderly’s independence, and an increase in their dependence on functions will be observed. ^3^ The elderly with dementia generally require a high level of long-term care. ^7^ These problems will lead to dependence and the urgent need for support by caregivers. ^8^ Approximately 85% of people with dementia are cared for by their families at home. ^9^ In many cases (57% -81%), women between the ages of 45 and 70 spend 12 hours daily taking care of a patient with dementia. Therefore, family caregivers of the elderly people with dementia are at high risk of losing mental and physical health. More than 80 percent of Alzheimer’s caregivers have experienced high levels of anxiety, ^10^ and about one third (25-30 percent) of dementia caregivers have reported clinical signs of anxiety. Anxiety accompanied by feelings of guilt and fatigue in caregivers that do not have adequate medical information about dementia and its usual symptoms and previous experience in how to care for these patients has been observed. Sometimes, caregivers often become anxious because they may not see the effects of their work or think that their care was not enough. ^11 - 13^ These harmful cases affect the well-being of the caregivers and also the ability to fully take care of the patient. ^8^

In addition, caregivers of the elderly people with dementia have a negative attitude in terms of physical health, energy, mood, memory and ability to perform recreational activities. ^14^ About half of the caregivers are suffering from high blood pressure. Arthralgia, obesity and limitation in activities, the prevalence of diabetes and high blood cholesterol in caregivers of the elderly people with dementia have also been reported. Reduction of the quality of life of caregivers reduces the quality of care for the elderly and has a negative effect on the progress of symptoms of dementia in the patient. ^15 , 16^

In order to prevent and reduce the negative side effects in caregivers of the elderly with dementia and to improve their health and quality of life, many studies have been conducted on the problems of caregivers and their lack of support, but the number of studies conducted for reducing anxiety of the elderly caregivers with dementia is limited. In addition, caregivers often have not received any formal education for dementia or for themselves. Since lack of adequate knowledge about illness and caring skills could lead to anxiety and exhaustion in caregivers and lack of enough knowledge of caregivers about self-care could also lead to physical and psychological complications and a decrease in their quality of life. ^8 , 16^ Therefore, the aim of this study was to determine the impact of on-site educational intervention along with informational support on the anxiety of family caregivers of elderly people with dementia.

## MATERIALS AND METHODS

This randomized, controlled trial was conducted from May to August 2017. The study was conducted in geriatric clinic in Imam Reza and other neurology clinics affiliated to Shiraz University of Medical Sciences, Shiraz, Iran. The target population included family caregivers of the elderly people with dementia living in Shiraz. Inclusion criteria for caregivers included being the patient’s main caregiver and taking on the main responsibilities of the patient; being over 18 years old; having reading and writing skills; having a three-month experience of patient care; access to telephone, mobile or social networks; and obtaining anxiety score more than 31 from Spielberger’s Anxiety Inventory and lower than 80.

The elderly person should also have a history of at least six months to five years of dementia. Exclusion criteria for the caregivers included getting physical and mental illness during the study which prevents patient care and drug abuse; using alcoholic drinks or psychotropic drugs; occurrence of a critical event or severe stress such as the death of closed ones, divorce; etc. during the study or in the last 3 months for the caregiver, participating of caregiver in training sessions on the care of the elderly people with dementia in the last 6 months; and taking care of someone else in the family. In addition, the death of the elderly person with dementia during the study, the history of the elderly in having other well-known chronic and debilitating diseases such as heart failure, uncontrolled asthma, uncontrolled diabetes, etc., before or during the study and transfer of the elderly patients with dementia to long-term care centers at the time of study were the other conditions of exclusion from the study.

In this study, the sample size was estimated according to the study carried out by Lynn et al. ^17^ For smaller effect sizes, we needed to enhance the sample size. Therefore, based on the following formula a 62-caregivers sample size was determined. Then, it was raised to 70 (35 subjects in each group).

$\mathrm{n1}=\mathrm{n2}=\frac{{\left(Z_{\mathrm{1 - \alpha ⁄ 2}}+Z_{\mathrm{1-\beta}}\right)}^{2}*s_{d}^{2}}{d_{2}}=35$

β=0.2-S_1_=7.16-S_2_=7.39-α=0.05-µ_1_=24.75- µ_2_=21.08

**Figure 1:**
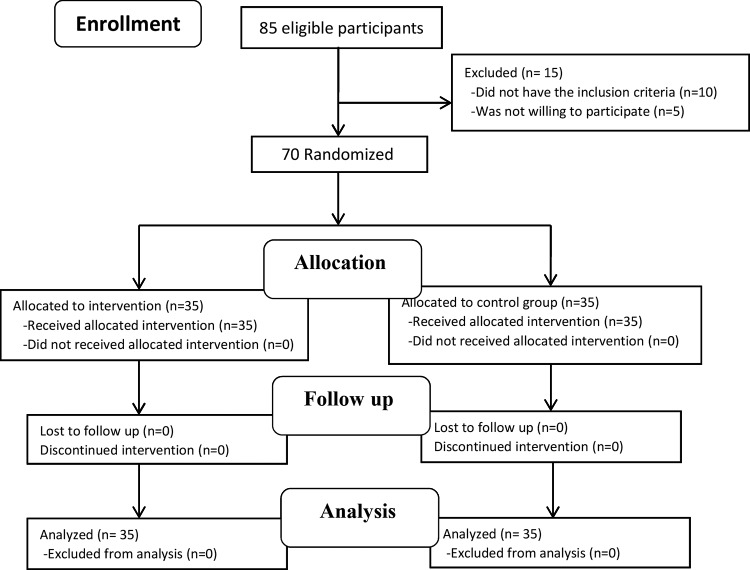
The flow diagram of the caregivers of the elderly with dementia through each stage of the study

The study participants were selected through convenience sampling when they referred to the neuropsychology and geriatric specialty clinic. Then, they were allocated to the intervention and control groups using block randomization with a random sequence of 2 block sizes. This was done through block design software. Each block was placed in a sealed package and sampling continued until the end. 85 caregivers were enrolled into the study. All subjects were invited to participate in the preliminary session to obtain their initial information and then, based on the inclusion criteria, they were evaluated, and eligible caregivers were selected. However, ten caregivers did not have the inclusion criteria, and five patients were not willing to participate in the study. Therefore, the study was performed on 70 subjects divided into intervention and control groups. It should be mentioned that all the 70 subjects finished the study (Figure 1). 

The intervention group was provided with educational intervention and a scientifically valid educational book approved by faculty members in Shiraz University of Medical Sciences. The control group did not receive any formal education regarding the care of the elderly with dementia, during the study. In order to have ethical considerations in the research, after conducting the study, the educational programs were carried out and also, a book was provided for them.

The caregivers signed written informed consents. Besides, they received verbal and written information about the study objectives, design, and duration. They were also reassured about their right to withdraw from the study. The confidentiality and anonymity of the caregivers’ information were considered, as well.

In this study, the assistant who collected the data and the statistician who analyzed the data were blind to the study groups. It should be noted that the caregivers in the intervention group were asked not to explain the program to other caregivers.

The study was approved by the Ethics Committee of the Shiraz University of Medical Sciences with the code of IR.SUMS.REC.1396.76.

The research included two stages of educational intervention and information stabilizing period. In the first stage, the way of caring an elderly with dementia and caring for her/himself were prepared in seven sessions of one and half hours and delivered by lectures and using educational aids such as PowerPoint, video projector, video player and educational imagery were taught for the intervention group (Table 1). Teaching special topics was done by senior lecturers, neurologists and researchers in each topic. The stabilizing of information provided by the researcher to the family caregivers of the intervention group during the 4-week training sessions, so that the researcher provided caregivers of the intervention group with the contact information and it was requested to make contact by telephone, SMS, video calling with virtual messaging programs or email three hours a day (from 18:00 to 21:00) and receive advisory services. If the patient required more specialized care, the caregiver was advised to refer to a specialist. 

**Table 1:** The contents of informational interventions in each session

Session No.	Educational caring type	Content of information	Teaching method technique
1	Patient care (elder with dementia)	General definition of the types of dementia	Discussion
2		Dementia treatments and drugs	Lecture, discussion, questioning strategies
3		Standard cares for the elderly with dementia	Lecture, discussion, questioning strategies
4		Physical and cognitive rehabilitation	Lecture, discussion, questioning strategies
5	Self-care (Caregiver)	Promoting adaptation and coping with the crisis situations and caring problems	Lecture, discussion, questioning strategies
6		Stress management methods	Role play, discussion
7		Promoting quality of life methods in family caregivers of elderly people with dementia	Discussion, questioning strategies, problem solving

After the end of the intervention (four weeks of educational intervention), the second stage of data collection was carried out by completing the Spielberger anxiety questionnaire by the caregivers of the intervention and control groups. Also, to assess the durability of the effect of education, three months after the intervention, the third stage of data collection from family caregivers in both groups was done. 

Demographic and Spielberger questionnaires were used in this study. The demographic questionnaire was self-made and included age , sex, marital status, etc. The State-Trait Anxiety Inventory (STAI) is a commonly used measure of trait and state anxiety. It can be used in clinical settings to diagnose anxiety and also often used in research as an indicator of caregiver distress. This questionnaire has 40 questions, 20 of which evaluate situational anxiety and 20 of them assess personality anxiety. The total score of each of the two scales of situational and personality anxiety is from 20 to 80. The scores of anxiety in Spielberger questionnaire are shown in Table 2. This tool is a valid questionnaire and has been used extensively in domestic and international researches. ^18^ Spielberger et al. (1970) state that for State Anxiety Scale, the stability coefficients range from 0.16-0.62, which are relatively low figures because a valid measure of State Anxiety Scale should reflect the influence of unique situational factors that may exist at the time of testing. The psychometric properties of the inventory in Iranian population were confirmed by exploratory and confirmatory factor analysis. The correlation between the inventory and clinical specialists’ assessment also showed appropriate validity. ^19^ The reliability of the Iranian form of the inventory was Cronbach’s alpha 0.88. ^20 , 21^

**Table 2:** The scores of anxiety in Spielberger questionnaire

Normal	<19
Mild	20-31
Moderate to downward	32-42
Moderate to upward	43-53
Relatively sever	54-64
Sever	65-75
Very severe	>76

Data collected from this study were analyzed by SPSS version 21 using descriptive (mean and frequency) and analytical statistics (ANOVA test with repeated data and independent t-test). 

## RESULTS

The normal distribution of variables was verified using Kolmogorov-Smirnov test. Findings showed that most of the family caregivers of the elderly in the intervention group 24 (68.6%) and control group 25 (71.4%) were females. Most of the caregivers 61 (87.1%) were children of the elderly persons in both groups. 24 (68.6%) caregivers in the intervention group and 29 (82.9%) in the control group were married. 29 (82.9%) of the subjects in the control group and 33 (93.4%) in the intervention group had a high school diploma and higher. 20 (57.1%) of the family caregivers of the elderly with dementia in the intervention group and 17 (48.6%) of the control group were employed and had an income.

There was no significant difference between the two groups in demographic characteristics of the caregivers of the elderly people (P>0.05). The mean age of the family caregivers was 46.8±9.9 years in the two groups. The mean age of elderly people was 73.6±6.96 years in the two groups and the mean duration of dementia in the elderly with dementia was 2.67±1.09 years. There was no significant difference in the age of caregivers (P=0.446), age of the elderly (P=0.425) and duration of dementia (P=0.069) in comparison of the two intervention and control groups. As a result, the two groups were identical regarding the age of the caregiver, age of the elderly and duration of dementia in them.

The mean anxiety scores of the family caregivers in the intervention group before the intervention, one and three months after the intervention, were 74.11±4.73, 70.51±3.78 and 70.31±3.43, respectively. Also, the mean anxiety score of the caregivers of the control group before and one and three months after the intervention were 75.63±3.08, 76.45±3.45 and 76.22±5.08, respectively; there was no significant difference in anxiety before the intervention in the two groups (Table 3).

**Table 3:** Comparison of the mean of anxiety score of the family caregivers of the elderly with dementia in the two groups of intervention and control before the intervention, one and three months after the intervention

Anxiety	Before the intervention Mean±SD	One month after Intervention Mean±SD	Three months after Intervention Mean±SD	*P value (within)
Intervention	74.11±4.73	70.51±3.78	70.31±3.43	<0.001
Control	75.63±3.08	76.45±3.45	76.22±5.08	0.14
P value (between)	0.12	<0.001	<0.001	

* ANOVA repeated measurements

**Figure 2:**
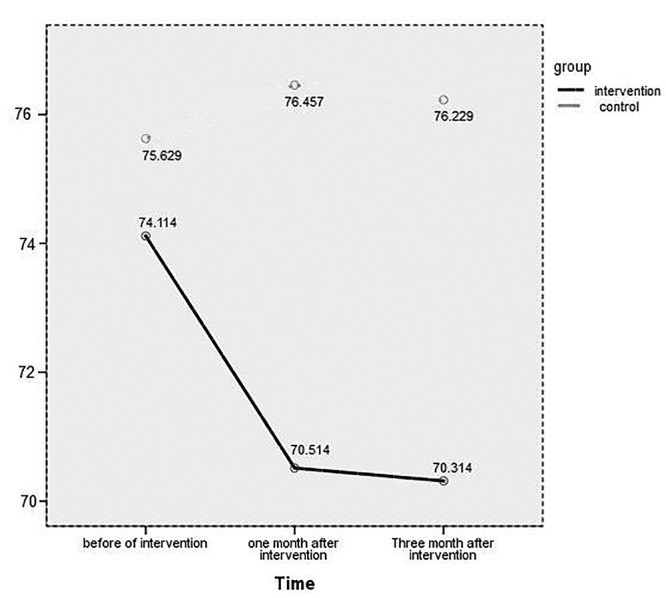
The mean changes in anxiety scores of family caregivers of the elderly patients with dementia before, one and three months after the intervention, by two groups of intervention and control

ANOVA test with repeated measurements showed that there was a significant difference between the two groups in terms of the anxiety mean score one month and three months after the intervention (P<0.05) (Table 3). This finding suggested that the reduction in anxiety in the intervention group was significant compared to the control group (Figure 2).

## DISCUSSION

The present study showed that the informational support of the caregivers of the elderly with dementia could reduce anxiety in caregivers. Education and care could reduce the anxiety of family caregivers of the elderly patients with Alzheimer’s disease. ^22^ Family education programs could improve the psychological situation of family caregivers of the elderly with dementia and reduce the caregivers’ anxiety. ^23^ Caregivers of the elderly patients with Alzheimer’s disease were not in a good general health and the educational counseling program could significantly improve the general health of the caregivers. ^24^ The family education program reduced anxiety in family caregivers of mental disorder patients and could potentially improve the quality of life of the caregivers and patients. ^25^ Also, this increased their satisfaction and awareness about Alzheimer’s disease, which reduced stress. Caregivers who had practiced different types of adaptive mechanisms during the educational intervention reported more favorable physical health and satisfaction. ^26^

Most caregivers were female and married. In Iran and in other countries, caring for chronic patients, especially the elderly, is done by women who are middle-aged and married. ^27^ In a study by the National Caregivers Union and the American Association of Retired Persons was found that more than half of the caregivers of patients with chronic disorders were middle-aged women with a mean age of 46 years. ^20 , 21^ These findings were in agreement with the present study. Previous studies have also shown that the less caregiver used adaptation methods to get adapted to the problems caused by illness, the more vulnerable they were to physical and psychological complications (anxiety and depression). ^28^

The results of the present study were in the same line with those of previous researches on the effectiveness of informational support on caregivers; however, what distinguished this study from other studies and made it unique was the existence of a non-intrusive in-person informational support program that was compiled and structured that caregivers, using easy and varied communication methods, shared care challenges at home with the researcher and surveyed the problems with the elderly care with the help of the researcher and at home to solve the problem. The use of various educational materials, such as using books and new and varied educational methods, in part of each session, was another advantage of this study. In this study and in most of the previous studies, the duration of the follow-up was short. Therefore, regarding the chronicity of dementia, family caregivers need longer support for the care of the elderly with dementia. Thus, considering the limitations and results of the study, it is suggested that the effect of more comprehensive psychological-spiritual programs such as emotional coping (dancing, deep breathing, yoga, relaxation, etc.) on the anxiety of caregivers should be investigated. It is also recommended to conduct more studies with larger sample size and longer follow-up time.

Due to the homogeneity of demographic data in both groups, these factors cannot be identified as confounding in this study. In addition, due to the generalization limitations of the results of the available sampling method, education and informational support is provided by the relevant organizations on a wider range of caregivers.

Nurses working in health centers, as well as sociologist nurses, other elderly people and home care centers need to receive the knowledge and skills necessary about threatening situations of the health of the elderly to maintain and promote the health and improve performance of caregivers and their elderly relative in all levels, especially family and community. 

## CONCLUSION

Anxiety among caregivers of the elderly with dementia is in high level, so according to the findings of this study, educational intervention can affect the family caregivers of dementia elderly and reduce their anxiety. Furthermore, many caregivers require more trainings and support as well as physical and mental health care. Therefore, provision of educational services for family caregivers of the elderly people with dementia is essential and should be specifically addressed in medical systems.
